# Medicine

**Published:** 1912-12

**Authors:** J. M. Fortescue-Brickdale


### Progress of the Medical Sciences.

### MEDICINE.

**The X-rays in abdominal conditions.** The responsibility for diagnosis in various abdominal conditions falls as often upon the physician as upon the surgeon, and consequently the recent discussion on the use of the X-rays in affections of the stomach and intestines at the French Surgical Association has considerable interest from a medical point of view. As regards **technique,** the general opinion was that carbonate of bismuth was safe and reliable; the sulphate of barium was also safe if chemically pure. Errors have arisen when the soluble sulphide was accidentally prescribed or dispensed. One speaker advocated oxide of zirconium, which, though only slightly opaque to the rays, can be given in very large quantities, as it is quite non-toxic. This substance ($Z_nO_2$) is insoluble except in hot concentrated sulphuric acid and hydrofluoric acid, which are not likely to be present in the stomach or intestines. According to Kaestle, it has greater absorptive power bulk for bulk than any other body, except thorium oxide, which is much more expensive. It is a tasteless fine white powder. The ordinary dose for stomach examination is two to three ounces. However, it costs 2s. 8d. an ounce, whereas bismuth oxychloride costs 10d., and pure precipitated barium sulphate 6d. Girard considers the lateral position of advantage in stomach examinations, and the Trendelenburg position for examination of the intestines.

Special points in **diagnosis** brought out by various speakers at the Congress were (*a*) elongation of the stomach occurs in cases of ptosis, owing to the fixation of the cardiac end and the partial fixation of the pylorus; the total length is sometimes as much as 30 c.m. (12 inches). (*b*) If the stomach is fixed by adhesions, the left side of the dome of the diaphragm is drawn up. (*c*) X-rays are of value in localising intestinal stricture, and in showing the fixation of the flexures of the colon by adhesions, as a result of chronic epiploitis. Fæcal concretions and atony of the gut are, however, very liable to be mistaken for organic stricture, and repeated examinations are necessary for correct diagnosis.

In a paper on the **diagnosis of ulcer of the stomach and duodenum,** Schutz summarises his experience of the value of X-ray findings. In functional disturbances the appearance of hour-glass contraction is often seen, but in these cases the situation and shape of the narrowed portion varies. In ulcer they are more constant, and especially when the ulcer is near the pylorus there is delay in emptying the stomach. In normal stomachs with good muscular tone a little bismuth often remains after six hours, but if any quantity is left behind, and the shadow extends to the left of the middle line, a spastic condition of the pylorus may be assumed, which is almost always due to ulcer. Often, on the other hand, when there are clinical signs of retention of food in the stomach, nothing can be seen on the screen after six hours. In fact, in simple ulcer of the stomach the X-ray findings are mostly negative. Chronic ulcers, with thickened edges, usually situated on the lesser curvature, appear in the X-ray picture as small masses of bismuth, directly continuous with the stomach wall, which disappear as soon as the main mass passes out of the organ. A perforating ulcer, which has formed a more or less marked cavity in a neighbouring part, shows a mass placed outside the stomach, often containing a small bubble of gas, and not disappearing when the stomach has emptied itself.

As perforating ulcers are often situated on the posterior wall, they are best looked for by transverse illumination. The change which this form often undergoes into a malignant ulceration cannot well be diagnosed by the X-rays. In carcinoma of the pylorus the stomach often empties itself rapidly, and the organ appears elongated, so that the pylorus takes the lowest position. On the other hand, in non-malignant ulceration the stomach empties slowly, and the pylorus is often brought nearer to the lesser curvature by adhesions. Although it is often impossible to determine by X-ray pictures whether a true hour-glass contraction of the stomach is due to malignant disease or to an old ulceration, the following points are sometimes of use. In ulcer the narrowing is nearer the pylorus, in cancer it is more median. In ulcer the narrowed portion is not so long as in cancer, and both sacs are usually the same size, whereas in cancer the lower one is the smaller.

Baron and Bársony have undertaken a systematic description of the X-ray findings in duodenal affections. The appearances investigated were (1) Drawing over of the stomach to the right which may involve the pyloric part only (pyloric dextro position), or the pyloric part and a portion of the upper segment of the stomach (total dextro position). (2) Hypertonus of the stomach. (3) Increased peristalsis, that is a rapid succession of peristaltic waves, which may divide the stomach into small round segments, when hypertrophy is present or into larger segments when it is absent. (4) The time occupied in emptying the stomach (5) Increased permeability of the pylorus. (6) More marked filling up of the duodenum. (7) The "nitch" symptom. (8) Circumscribed areas of increased sensitiveness to pressure localised by the X-ray shadow.

The authors come to the following conclusions. (1) The "nitch" symptom is the only certain sign of duodenal ulcer; it is that appearance already described as occurring in perforating gastric ulcer. In duodenal cases carcinomatous ulcers, simple ulcers and diverticula cannot by this means be differentiated. (2) Organic stenosis of the duodenum can be diagnosed with certainty if Holzknecht's syndrome is present, namely: the duodenum is distended above the stricture, and the distension persists on account of the ineffective peristalsis. Remains of the bismuth meal are seen above the stenosis, and the dilatation may extend to the stomach, especially if the stricture is high up. Spastic contractions produce the same effect, but if the condition is slight and localised to the site of the ulcer the appearances will be less marked, only a little bismuth will remain just above the contraction after six hours. There is, moreover, no dilatation of the duodenum and the shape of the stomach does not undergo changes. Incompetence of the pylorus is present in duodenal stenosis. Reversed peristalsis may be seen. (3) In stenosis compensated by hypertrophy the syndrome does not appear. (4) X-rays cannot show whether the stenosis is due to lesions in or outside the duodenum. (5) Slight degrees of stenosis in the vicinity of the pylorus present great difficulties of diagnosis. Simple ulcers without stenosis produce the same effects, namely complete filling of the duodenum above the lesion, increased permeability of the pylorus, and increased peristalsis of the stomach. If some bismuth remains in the stomach or duodenum after six hours, a spasmodic contraction of the latter owing to ulcer may be suspected. (6) The shape and time of emptying of the stomach may be normal even in extreme degrees of stenosis. (7) The "nitch" symptom is the only certain sign of non-stenotic lesions of the duodenum. In its absence, an hour-glass contraction of the stomach which has been proved to be spastic and not organic in origin may show either a single duodenal or a single gastric ulcer. In favour of the former are increased permeability of the pylorus, increased peristalsis confined to the lower segment of the stomach, which is completely emptied in six hours, and a circumscribed point of tenderness corresponding to the projection of the duodenum. The reverse is true for gastric ulcer. Both, however, may be present at the same time, and then the X-ray picture may correspond to that of the duodenal lesion. The same picture without change in the shape of the stomach or duodenum makes a diagnosis of duodenal ulcer probable. (8) It is impossible to diagnose between ulceration of the pylorus and of the neighbouring parts of the stomach and duodenum in the absence of increased permeability and rapid peristalsis. (9) A high degree of duodenal stenosis causing dilatation of the stomach cannot be diagnosed. (10) In many cases of ulcer the X-ray picture is normal. (11) In cancerous achylia and cancerous infiltration of the pylorus, the pylorus may be more permeable than normal and the stomach apparently hypertrophied. Diagnosis is impossible when the characteristic duodenal signs are absent. (12) The greatest difficulty occurs in the differential diagnosis between duodenal lesions and those of the gall-bladder. Even the "nitch" may be produced in the latter. Operation may fail to make clear the primary lesion.

Kretschmer has investigated the effect of the **dietary treatment of constipation** by means of the X-rays. His first experiment was as follows. The patient was given barium sulphate made into a purée, and then fed for a day and a half on a bland diet, consisting of eggs, milk, gruels, white meat, soup, white bread and butter. Radiograms were taken at intervals of six, twenty and thirty hours after the barium sulphate had been given. His second experiment was similar, but the diet consisted of food calculated to stimulate peristalsis, namely cabbage and other fresh vegetables, fruit, jams, buttermilk and sour milk. The main differences in the position of the barium sulphate were seen during the first six hours, that is in the small intestine and first part of the colon. With stimulating diet the barium had reached a point in six hours which it took twenty hours to reach on bland diet. After thirty hours the difference was only very slight. The influence of diet, therefore, on chronic constipation seems to be limited to its effect in increasing peristalsis in the small intestine and the first part of the colon.

Direct **medication of the duodenum** by means of Einhorn's sound is the subject of a paper by Skaller. By means of X-ray demonstrations he shows that in order that the sound may enter the duodenum it is necessary that the patient should lie down, so that the weight of the capsule should not cause it to gravitate to the lowest point of the stomach. It is also necessary that the stomach should be quite empty. According to the patient's height and the degree of gastroptosis present (if any) 50 to 70

c.m. of the sound are swallowed, and after ten to fifteen minutes' interval another 20 c.m., the patient lying on the right side. The sound reaches the duodenum in about twenty-five minutes from the beginning. The process is quite painless. Skaller does not think the X-ray picture alone capable of demonstrating the presence of the capsule in the duodenum. It is often difficult or impossible to see it, and impregnating the tube with bismuth is of little aid. The X-rays are very useful, however, in conjunction with three other tests. The first of these consists in aspirating some of the contents of the viscus. In the duodenum they should be alkaline and bile stained. Sometimes, however, they may be neutral, and sometimes nothing can be withdrawn. At other times the duodenal contents may regurgitate into the empty stomach, and render the test unreliable. The second test consists of introducing air into the bowel. If the capsule is in the stomach the patient is aware of the introduction of the air, if it is in the duodenum he fails to feel anything. Percussion will confirm this. The third test consists in auscultating the abdomen while air is being introduced. The inrush of air is best heard to the left of the middle line if the capsule is in the stomach, and to the right if it is in the duodenum. Borborygmi can also be heard immediately in the latter case, but not in the former. If all these tests agree, the position of the capsule is certain.

J. M. FORTESCUE-BRICKDALE.

REFERENCES.

[1] XXV Congrès de l'Association Française de Chirurgie, *Gaz. d. Hôp.*, 1912, lxxxv. 1640.

[2] Kæstle, *München. Med. Wchnschr.*, 1909, lvi. 2576.

[3] Schütz, *Wein. Klin. Wchnschr.*, 1912, xxx. 1519.

[4] Baron and Bársony, *Ibid.*, p. 1521.

[5] Kretschmer, *München. Med. Wchnschr.*, 1912, lix. 2334.

[6] Skaller, *Berl. Klin. Wchnschr.*, 1912, xlix. 2131.

